# Supplementary material for: Acceptability testing of the Carers-ID intervention to support the mental health of family carers of people with profound and multiple intellectual disabilities
Source: PLoS One. 2024 Oct 31;19(10):e0313081. doi: 10.1371/journal.pone.0313081 (PMC11527193; doi:10.1371/journal.pone.0313081)
Supplement: S1 Table — (DOCX) [file pone.0313081.s001.docx]

| **Factor** | **Mean (SD)** | **Median (IQR)** |
| --- | --- | --- |
| Male | 72.77 (10.03) | 72.50 (18) |
| Female | 76.65 (9.89) | 78 (19) |
| Mother | 75.50 (9.82) | 77.50 (17) |
| Father | 70.94 (9.91) | 70.50 (17) |
| Grandparent | 84.67 (5.77) | 88 (.) |
| England | 76.41 (9.87) | 78 (21) |
| Scotland | 73.89 (11.26) | 74.50 (21) |
| Wales | 76.42 (10.51) | 77.50 (22) |
| Northern Ireland | 73.67 (9.71) | 77.50 (14) |
| Number in family (n = 2) | 72.20 (12.03) | 74 (23) |
| Number in family (n = 3) | 76.04 (9.47) | 78 (16) |
| Number in family (n = 4) | 75.57 (10.77) | 75 (21) |
| Number in family (n = 5) | 72.50 (5.92) | 72 (11) |

**S1 Table**

Mean (standard deviation) and median (interquartile range) for sex, carer, carer family size, and country of residence, on programme acceptability total score.
