# Supplementary material for: Acceptability testing of the Carers-ID intervention to support the mental health of family carers of people with profound and multiple intellectual disabilities
Source: PLoS One. 2024 Oct 31;19(10):e0313081. doi: 10.1371/journal.pone.0313081 (PMC11527193; doi:10.1371/journal.pone.0313081)
Supplement: Attachment — Submitted filename: Response to Reviewers.docx [file pone.0313081.s002.docx]

**Response to Reviewers**

| **Journal requirements** | **Author response** |
| --- | --- |
| Please ensure that your manuscript meets PLOS ONE's style requirements, including those for file naming. | We have reviewed our manuscript and amended all format where necessary.  We have adhered to all file naming guidelines and formatting guidelines. |
| Please state what role the funders took in the study. | We have amended the financial disclosure statement. |
| We note that you have provided funding information that is currently declared in your Funding Statement. However, funding information should not appear in the Acknowledgments section or other areas of your manuscript. We will only publish funding information present in the Funding Statement section of the online submission form. | We have removed financial information from the acknowledgements and amended this section. |
| We note that this data set consists of interview transcripts. Can you please confirm that all participants gave consent for interview transcript to be published?  If they DID provide consent for these transcripts to be published, please also confirm that the transcripts do not contain any potentially identifying information (or let us know if the participants consented to having their personal details published and made publicly available). | We confirm that all participants consented to their anonymised data being published.  We have added a data availability statement, as all data associated with this publication is openly available in UK data service ReShare. |
| **Reviewer 1 comments** |  |
| This study is an excellent example of how qualitative data when properly integrated and mapped can provide comprehensive pathway in understanding unique lived experiences. Moreover, the study further demonstrates value of mapping individual experiences into a wider context and how such approaches can support development of tools that can be easily accessible for target population to 1) prepare 2) support and 3) guide the experience. | No response required. |
| **Reviewer 2 comments** |  |
| The paper describes the acceptability testing of the Carers ID intervention to support the mental health of family carers of people with profound and multiple intellectual disabilities. I found the paper easy to read and quite straightforward in the flow through the story of the paper. I appreciate the truly mixed methods approach to the assimilation of the qualitative and quantitative data - this is refreshing to see within studies and not often approached this robustly. On the whole I liked this manuscript and would support the publication of this. | No response required. |
| It is noted that the acceptance scale has been adapted from another measure. Whilst some psychometrics are provided for this adaptation the description and process of this adaptation requires further justification given that it looks like these values have been calculated on the collected data that is to be analysed. Confirmation of data structure and measure validity would have been better conducted on an independent data set and possible introduction of any bias should be considered by conducting confirmatory psychometric analysis alongside the analysis of the data. | Only minor wording was changed from the original scale, we did not change the overall structure of the scale and therefore a psychometric testing was not deemed necessary.  We have added some further information to clarify this. |
| Participants are rewarded for their time to the tune of £10 and £20 - it is not clear when these are received or in what they relate to in terms of the timeline. For reproducibility it would be better to understand the flow of these rewards within the research scope and timeline. | Further detail has been added to clarify who received and when the voucher were received. |
| The data analysis section is worded strangely - "descriptive statistics were used to calculate means and standard deviations for each subscale and a total score on the AHAA" This implies that subscales and scores were broken down by descriptive categories (which are not presented). The description of analysis could be clearer. | We have revised to make this clearer. |
| "Participants could score a minimum of 22 or a maximum of 88 with higher scores indicating a greater degree of acceptability" This sentence was repeated 3 times in the manuscript, I would suggest avoiding repetitive use of information. | We have removed this from the results to avoid repetition. |
| Sample size justification - there was no justification that the sample size for the quantitative elements were adequate. Given the design there may not have been a power calculation as such but there should be some justification of the sample aimed for or of that captured in the ability of the sample to answer the research aims and objectives. | It is important to note that we are not aiming to determine an effect with this study therefore a power analysis is not appropriate. This is an exploratory study.  However the authors recognise that some justification for our sample size would be useful. We have added some further detail. |
| Table 4 provides some of the descriptive information of the measures - It appears there may be some skew within the data (and possibly ceiling effects on at least the first three subscales), therefore I wonder would it be more appropriate to include median and IQR as well, ensuring that the data is being represented accurately. | We have now added medians and IQR ranges for all subscales and total score. |
| I'm not convinced of the added value of the t-tests conducted - what were the hypothesis here, this was not outlined as part of the original research questions or part of the aims and objectives and with no justification on sample achieved it is difficult to tell if these would be expected findings or those generated by a small sample, particularly given the imbalance seen within some of the groups. Additionally, given the potential skew seen in the data did the required assumptions for the tests hold? Possibly providing the breakdown of the scores by comparison group in a supplementary table would enable the readers to understand the data presented more thoroughly. | We take on board the reviewers comments that given the small sample size, the t-test preformed may have limitations. However, this is an exploratory study of acceptability to which the t-tests and comparison of scores given some basis for further exploration. We have addressed some of the issues with the sample in our limitations, but believe that the triangulation of quant and qual findings strengthen the findings further.  We have also added supplementary data which presented a descriptive comparison of scores between the groups analysed. |
| Finally, there still appears to be some question over the effectiveness of this intervention. I acknowledge that this was not an aim or objective of this piece of research but given the findings I think it would be useful in the discussion to expand upon future plans for understanding the effectiveness of the intervention and what that may entail. | There are some findings in relation to carers perceived effectiveness, specifically that the intervention may be more effective for those at the beginning of their caring journey. This is discussed in depth in the discussion.  We did not measure effectiveness of the intervention in this study so therefore cannot elaborate further than we already have. |
