# Supplementary material for: Acceptability testing of the Carers-ID intervention to support the mental health of family carers of people with profound and multiple intellectual disabilities
Source: PLoS One. 2024 Oct 31;19(10):e0313081. doi: 10.1371/journal.pone.0313081 (PMC11527193; doi:10.1371/journal.pone.0313081)
Supplement: Attachment — Submitted filename: Response to Reviewers 12.09.24.docx [file pone.0313081.s004.docx]

**Response to Reviewers**

| **Journal requirements** | **Author response** |
| --- | --- |
| The ethics statement should note on the manuscript that informed written consent was gathered. | This has now been added. |
| The limitations should consider the small sample size for the quantitative work, and any limitations with confounding in analysis. | We have now added this to the limitations section. “In the quantitative phases the small sample size limits the generalizability of the findings. However, through our recruitment efforts we made every attempt to make sure the sample was diverse and reflective of the carers population as possible.” |
| **Reviewer 2 comments** |  |
| Minor comment "Participants who completed the survey received a £10 voucher following competition." - think this should read completion. | We have now corrected this error. |
| Apologies if I had not made my comment previous comment clear "Finally, there still appears to be some question over the effectiveness of this intervention. I acknowledge that this was not an aim or objective of this piece of research but given the findings I think it would be useful in the discussion to expand upon future plans for understanding the effectiveness of the intervention and what that may entail." I understand that assessing effectiveness was not an aim of the study however, I wondered whether in the discussion it would be useful to include what future plans would be for establishing effectiveness of the intervention given you have established acceptability here but this would be merely a preference inclusion rather than a requirement. | Thank you for clarifying this point, we have now added the following to the discussion: “In addition, given that this current study has established acceptability, future research should now examine the feasibility and effectiveness of the Carer-ID intervention”. |
